# A New Synopsis of Nosology, Founded on the Principles of Pathological Anatomy, and of the Natural Affinities of Diseases

**Published:** 1834-07-01

**Authors:** 


*A New Synopsis of Nosology, founded on the Principles of Pathological Anatomy, and of the Natural Affinities of Diseases.* By G. HUME WEATHERHEAD, M.D.—London, 1834. 18mo. pp. 90, and xxix.

DR. WEATHERHEAD says, in his introductory remarks,

"The author has found that he has been able, by following the natural affinities, to classify all the diseases resulting from the derangements proper to the body, spontaneous and incidental, under four principal heads. These form his classes: namely, the Phlogotici, or pure inflammatory diseases; the Hæmapharmatici, or those diseases which originate from a miasm, or poison, entering the healthy blood, and tainting it; thirdly, the Neurotici, which comprehend all those diseases arising from affections of the nervous system, unaccompanied by any apparent structural disorganization; while the last class, or the Vitia, comprises all accidental disfigurations, new morbid formations, extraneous lodgments, and congenital malformations." (P. xvii.)

Now, if for this barbarous term, Hæmapharmatici, we substitute Cachexiæ, and say Locales instead of Vitia, we shall have Cullen's four classes, which we prefer with the original names. In the various subdivisions our author is so much indebted to Dr. Young, that we could have wished to see some acknowledgment of his obligations. Surely there is a strong resemblance between the following species of Vulnus, as given by the two writers:

"Vulnus (a wound). α. Simplex. β. Laceratum. γ. Puncturatum. δ. Penetrans. ε. Contusum. ζ. Venenatum." (P. 73.)

"V. simplex. V. penetrans. V. laceratum. V. contusum. V. ablatitium. V. venenatum." (*Young's Medical Literature*, p. 407, 2d edit.)

Dr. Weatherhead does not follow the ancients in the genders of his words: thus, at p. 74, he makes *ulcus* masculine, and, at p. 82, *pes* feminine; but, at p. 81, this bold innovator ventures on a still more violent infringement of the *propria quæ maribus*, and actually makes *penis* feminine! Then, at p. 81, we have a mistake of the kind for which boys get their ears boxed at all well-conducted schools: *cor expers* is used to signify "heart wanting," *vesica expers* a "deficiency of the bladder," and so on. At p. 22, Dr. Weatherhead says, that *phrenitis* "is obviously a very improper name for any disease; since, according to the pathological meaning now attached to *itis*, it signifies *inflammation of the mind*." Poh! poh! Phrenitis signifies inflammation of the brain, and is one of the best names we have; for it is not one of the rickety, make-believe Greek words, manufactured by dictionary-hunters, but a genuine long-lived term, as old as the hills, and likely to last as long.

Yet we would not have our readers suppose that our author, though unskilled in verbal niceties, is altogether deficient in ingenuity; far from it. We approve, too, of his giving after each disease the usual sequelæ; and the following passage may show that, if Dr. Weatherhead will give up the arid study of nomenclature, in which he is far from happy, he may be infinitely more successful in other branches of medical knowledge.

"The pathology of jaundice, as at present laid down in books, is that it arises from the reabsorption into the circulation of bile that had already been separated from the blood, from an impediment existing somewhere to the transit of this fluid into the duodenum. Now, without insisting on the prodigious quantity of bile that would be required to tinge yellow so large a mass of an already deeply coloured fluid as the blood, it may be allowed us, in this place, to refer to the experiment of M. Magendie, who, when he injected only about two drachms of bile into the veins of an animal of the middle size, caused its death. Again, it may be asked, how comes it so frequently that jaundice shall occur, and yet no impediment to the free issue and discharge of the bile shall be found to have existed? Another circumstance deserving of recollection is, that bile is often seen to be separated in abundance from the blood by the kidneys, for several days *before* any jaundiced yellowness is perceptible even in the conjunctiva. And the last fact I shall notice, subversive of the doctrine of reabsorption, or of the necessity for it at all, is, that the experiments of Orfila, Chevreul, and Clarion, have established, that several of the immediate principles of bile, if not bile itself, exist naturally in the blood; and hence it would appear, that the office of the liver in separating this fluid from the circulation is not formative, but secretory, in the most literal meaning of the word.

"Premising these few remarks, we now can see why and in what manner jaundice may be produced, without having recourse to the improbable doctrine of reabsorption, against which a strong array of other circumstances militates. The liver ceasing its function of separation from any cause, the bile, or its immediate principles, thus accumulate in the blood; and, when this takes place, the author conceives several vicarious actions are instituted as suppeditories: the kidneys are made to strain off a large quantity of it; cases are on record where the salivary glands have secreted it in abundance; and the yellowness of the skin, he conceives, is entirely owing to the same suppeditory function being instituted by the exhalants that open on the surface of the body: hence we not unfrequently see the body-linen of a jaundiced patient tinged yellow." (P. 67.)

